# Breathing of the Nevado del Ruiz volcano reservoir, Colombia, inferred from repeated seismic tomography

**DOI:** 10.1038/srep46094

**Published:** 2017-04-10

**Authors:** Carlos. A. Vargas, Ivan Koulakov, Claude Jaupart, Valery Gladkov, Eliana Gomez, Sami El Khrepy, Nassir Al-Arifi

**Affiliations:** 1 Universidad Nacional de Colombia, Department of Geosciences, Ciudad Universitaria, Bogota, Colombia; 2 Trofimuk Institute of Petroleum Geology and Geophysics, SB RAS, Prospekt Koptyuga, 3, 630090, Novosibirsk, Russia; 3 Novosibirsk State University, Novosibirsk, Russia, Pirogova 2, 630090, Novosibirsk, Russia; 4 Institut de Physique du Globe de Paris, Sorbonne Paris Cité, CNRS (UMR 7154), 1 rue Jussieu, 75238 Paris, Cedex 5, France; 5 King Saud University, Riyadh, Saudi Arabia, P.O. Box 2455, Riyadh, 11451, Saudi Arabia; 6 National Research Institute of Astronomy and Geophysics, NRIAG, 11421, Helwan, Egypt

## Abstract

Nevado del Ruiz volcano (NRV), Columbia, is one of the most dangerous volcanoes in the world and caused the death of 25,000 people in 1985. Using a new algorithm for repeated tomography, we have found a prominent seismic anomaly with high values of the Vp/Vs ratio at depths of 2–5 km below the surface, which is associated with a shallow magma reservoir. The amplitude and shape of this anomaly changed during the current phase of unrest which began in 2010. We interpret these changes as due to the ascent of gas bubbles through magma and to degassing of the reservoir. In 2011–2014, most of this gas escaped through permeable roof rocks, feeding surface fumarole activity and leading to a gradual decrease of the Vp/Vs ratio in the reservoir. This trend was reversed in 2015–2016 due to replenishment of the reservoir by a new batch of volatile-rich magma likely to sustain further volcanic activity. It is argued that the recurring “breathing” of the shallow reservoir is the main cause of current eruptions at NRV.

Nevado del Ruiz (NRV), an active stratovolcano culminating at an altitude of 5,321 meters in Colombia (Fig. 1), is located in a highly populated area, which makes it one of the most dangerous volcanoes of the world. An eruption of moderate volcanic explosivity index (VEI) between 2 and 3, on November 13, 1985, triggered a large lahar that destroyed the city of Armero ~30 km away from the summit, and killed 22,000 people^1^,^2^,^3^. It also affected the populations of Chinchina and Rio Claro causing approximately 3,000 fatalities. This event is thought to be the deadliest recorded lahar in human history. Nevado del Ruiz has been active through the Quarternary^4^, with at least three VEI-4 eruptions in the past 2,000 years, in 1350 AD, 200 BC and 850 BC^5^. An eruption of similar magnitude would cause the melting of the massive ice sheet that caps the volcano and would generate larger lahars than that of 1985 with catastrophic consequences for the area.

Beginning in 2010, NRV has been in a phase of volcanic unrest with intense seismic activity, surface deformation and gas venting. The SO_2_ flux reached extreme values of 30 ktons per day at times and the cumulative output of SO_2_ from 2012 to 2015 is estimated to be 7 10^6^ tons^6^ (Extended Data Figure S1). We estimate that the flux of H_2_O and CO_2_ exceeds this value by a factor of at least 10 (Supplementary material).

Following the catastrophic 1985 event, Colombian authorities have made important efforts to improve the monitoring of volcanic activity at NRV through the deployment of a large number of instruments. Inclinometers have revealed large amounts of surface deformation close to the main Arenas crater between May 2012 and the present^6^. Deformation climaxed with a lava dome eruption in the eastern sector of the crater from September to November 2015^6^. Beginning in 2015, drumbeat seismicity, a long series of events repeating themselves at regular time intervals with identical waveforms, has been recorded in the crater area^7^. Ground inflation over a broad area has been occurring at a rate of ~4 cm per year^8^,^9^ with a deformation center that lies ~10 km to the south of the NRV edifice at a depth of ~14 km^8^.

Since the 1980s, NRV has been monitored by a permanent seismic network that has been expanded gradually. Data from this network have been used to determine the locations of seismic events, their spectral characteristics^10^, time changes of seismic attenuation beneath the volcano^11^ as well as a three-dimensional seismic crustal model^12^. Compared to this model, which was derived from body-wave data recorded before 2002, the present work yields important new information. The extensive data set that has been acquired since 2002 allows large improvements in the resolution of tomographic inversions. Furthermore, the seismic velocity structure of NRV has changed considerably as a result of volcanic and magmatic activity, especially during the latest phase of unrest that began in 2011. Using large amounts of data over a long time interval and a new algorithm for repeated tomography, we have derived a new detailed 3-D seismic model and have determined changes of seismic velocity beneath the volcano.

## Repeated tomography

In recent years, several authors have investigated time variations of seismic structure beneath active areas using repeated tomographic inversions^13^,^14^,^15^. Results are typically derived from the same calculations performed over different time intervals and hence may be affected by artefacts due to changes of data coverage. An algorithm was developed specifically to overcome this problem^16^ based on a selection process that generates data sets with similar data distributions. This algorithm was only suited to dense seismic networks and a homogeneous spatial distribution of seismicity and we have modified it to handle data from “non-ideal” networks, as is often the case in volcanic areas. The basic method is as follows. We consider a pair of datasets from two different time intervals. For each event in the first dataset, we look for a “paired” event in the second dataset with a maximum number of common phases recorded at the same stations. This yields two datasets with similar ray paths, which effectively minimizes data coverage variations. Here, this methodology is applied to volcanic areas for the first time. Details on the algorithm may be found in the Supplementary material. The complete code is available online together with data from this study, allowing all interested parties to reproduce our calculations.

To reveal changes of velocity structure beneath NVR, we have focussed on three pairs of time intervals (2011–2012 vs. 1998–2010, 2011–2012 vs. 2013–2014, and 2011–2012 vs. 2015–2016) that were selected according to the amount of data available and features of the volcanic activity. The same interval (2011–2012) was taken as reference in all cases. For each pair of time intervals, we sought distributions of stations, events and ray paths that were as close to each other as possible (Fig. 2), which led to three different data sets for the reference time-interval. We were not able to achieve totally identical distributions but checked that the impact on inversions results was small. This was achieved by comparing the three different velocity models obtained for the reference time-interval.

The ability of the inversion algorithm to resolve time changes of seismic structure was assessed using a series of synthetic tests (Supplementary materials). For example, starting from the same synthetic model (e.g., a checkerboard in Extended Data Figure S3), we found that changes of seismic ray coverage lead to differences of velocity values that are much smaller than those obtained from the experimental data. In a second series of tests, we used synthetic anomalies with more realistic shapes (Extended Data Figure S4). The algorithm was able to retrieve changes of seismic anomalies that are close to those obtained from the experimental data. More details on the synthetic modeling are given in Supplementary Materials.

## Time changes of seismic structure beneath NRV

**Figure 1:**
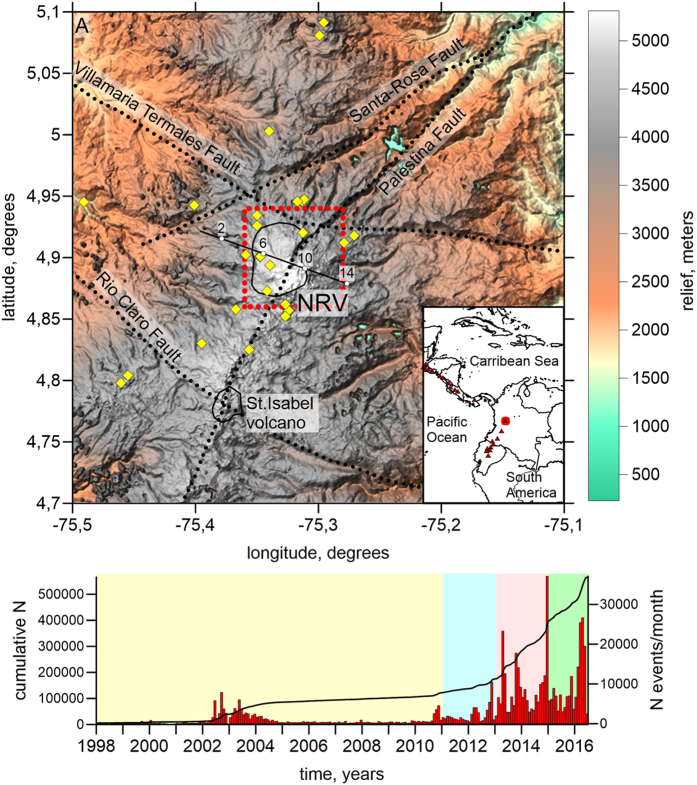
Study region and data information. (**A**) The topography of study area with main tectonic faults indicated (black dotted lines). Yellow diamonds depict seismic stations used in this study; black line indicates profiles used for showing the main results; red dotted line highlights the high-resolved area for which resulting maps are presented; solid black lines highlight major volcanic complexes in the area. The inset shows the location of study area (red dot); red triangles depict active volcanoes. NRV is Nevado del Ruiz Volcano. (**B**) Numbers of events per month and cumulative value for dataset used in this study. Areas of different colors highlight the time periods used for data selections. This picture is produced using Surfer 12, Golden Software.

**Figure 2:**
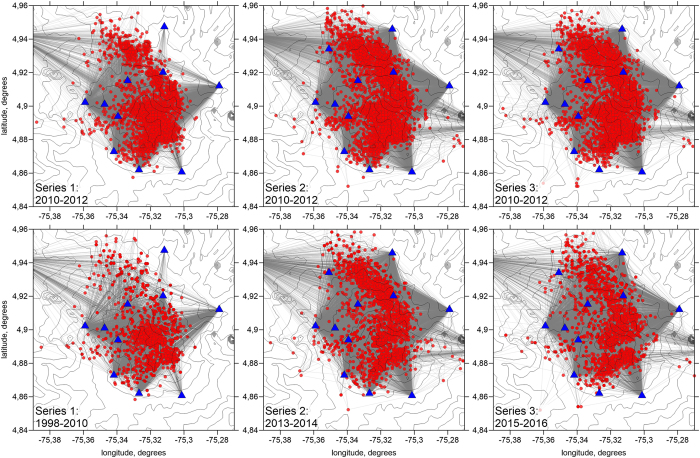
The distributions of data in two inversion series. The gray lines depict the paths of the P-rays; red dots are the selected events for the specific interval, and blue triangles are the seismic stations provided the data for the current subset. The contour lines depict the relief. This picture is produced using Surfer 12, Golden Software.

**Figure 3:**
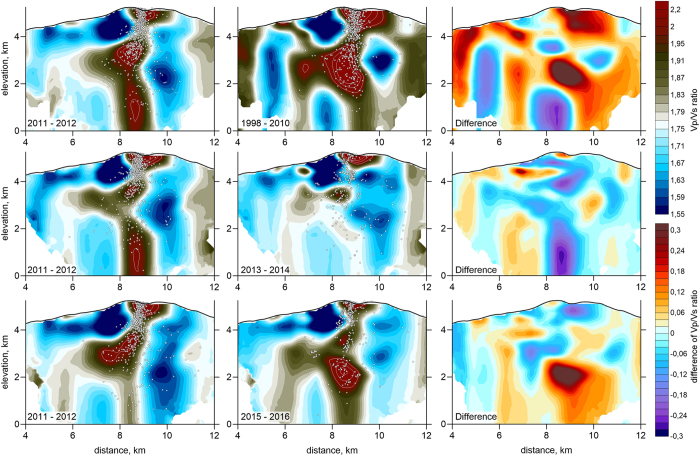
Results of repeated tomography inversions in the vertical profile indicated inFig. 1. Each row shows the resulting *Vp/Vs* ratios and their differences corresponding to two time intervals in one of the three series. Dots depict events located at distances of less than 0.4 km from the profile. This picture is produced using Surfer 12, Golden Software.

Figure 3 shows the distributions of *Vp/Vs* ratios in a vertical cross-section through the NRV summit (Fig. 1) for the three paired data sets. The distributions of *P* and *S-*wave velocities and *Vp/Vs* ratios in other vertical and horizontal sections can be found in Extended Data Figures S5–S10. The major feature of our velocity model is a prominent high *Vp/Vs* anomaly beneath the volcano summit. For the period 1998–2010, the *Vp/Vs* ratio is larger than 2.2 due to *P* and *S* velocities that are higher and lower than normal, respectively (Extended Data Figure S5). Similar characteristics have been observed in other active volcanoes^14^,^17^. *P*-wave velocity is sensitive to composition, whereas *S*-wave velocities are mostly affected by the presence of liquid or gas phases (volatiles or melts)^18^,^19^. Therefore, the coexistence of higher *P* and lower *S* velocities is often interpreted as due to magma with a more primitive composition (higher *P*) that is saturated with volatiles and carries a gas phase with a small crystal load (lower *S*). At NRV, the anomalous high *Vp/Vs* region is associated with high seismicity, which can be attributed to fracturing due to high pressure in the magma reservoir and in the permeable roof rocks.

The most striking observation is a gradual decrease in amplitude and size of the *Vp/Vs* anomaly in 2011–2014 (Fig. 3). The anomaly almost disappears in 2013–2014 and becomes prominent again in 2015–2016 at about the same location with almost the same amplitude as the initial one.

## Migration of volatiles beneath NRV

**Figure 4:**
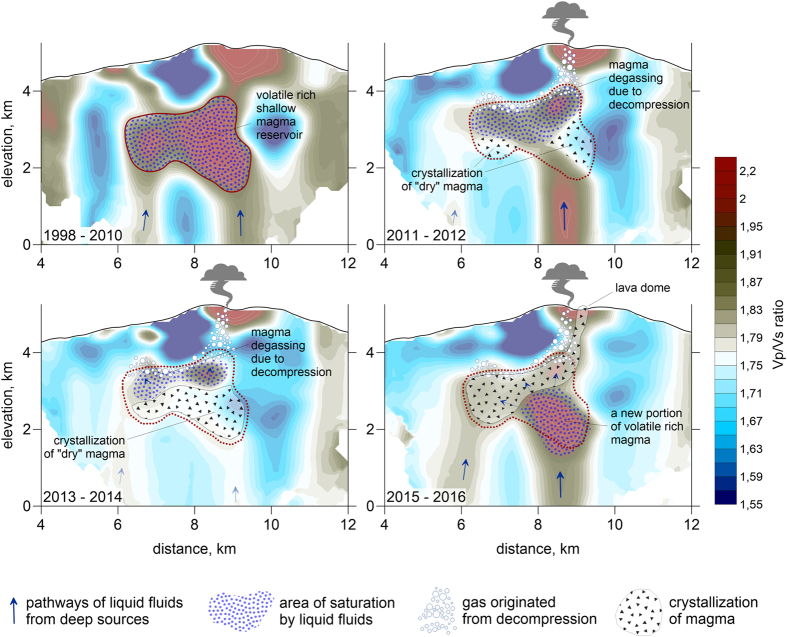
Stages of magma reservoir development according to our repeated tomography. The background is the distribution of *Vp/Vs* ratios in four time intervals, (the same as that shown in Fig. 3). This picture is produced using Surfer 12, Golden Software.

Figure 4 shows our interpretation of the seismic results. The high *Vp/Vs* anomaly beneath the volcano is attributed to volatile-rich magma in a shallow reservoir. The upper boundary of the anomaly lies at ~2 km depth and remains at the same location independently of the velocity changes that occur. This boundary is interpreted as the reservoir roof. In marked contrast, the lower boundary of the anomaly moved upwards in 2011–2014. We propose that the relatively rapid changes of *Vp/Vs* are due to the migration of fluid/gas phases within the reservoir.

The large amounts of SO_2_ gas that were emitted by NRV in 2012–2016 are not exceptional and many other volcanoes exhibit the same behaviour^20^. It has proven impossible to reconcile the sulfur and magma budgets of these volcanoes without the presence of a SO_2_ gas phase at great depths^21^,^22^. In such conditions, magma storage at shallow depth allows the escape of large amounts of gas. Owing to its low concentration, SO_2_ does not account for a large volume fraction in magma even at shallow depth and it is the exsolution of the more abundant H_2_0 that promotes significant gas volumes^20^.

At NRV, there was only mild fumarolic activity in the initial time-interval when the *Vp/Vs* ratio was largest, suggesting that the seismic anomaly was due to volatile-saturated magma and gas in the reservoir. Melt inclusion and glass data support this interpretation^20^. The water content of volatile-saturated melt is dictated by solubility and varies as a function of pressure and CO_2_ content. The lowest glass water content of 1.6 wt% provides an estimate of the shallowest storage depth, which must be at least 0.8 km^23^. Similarly, the largest water content of 3.3 wt% indicates a minimum depth of 3.1 km^23^. These two different estimates may be interpreted as indicative of the thickness of the shallow NRV reservoir and are are consistent with the seismological evidence.

From 2012 to 2014, the *Vp/Vp* seismic anomaly gradually waned as large amounts of gas were vented from the volcano. The total mass of SO_2_ gas emitted was 7 × 10^6^ tons, which allows an estimate of the associated magma volume (Supplementary material). We find volumes in a 1–5 × 10^9^ m^3^ range, corresponding to an average diameter between 1.4 and 2 km for a spherical reservoir, close to the dimensions of the seismic anomaly. Gas venting must be fed by gas bubbles rising through magma, which implies the growth of a degassed region at the base of the reservoir. This is consistent with the shallowing of the high *Vp/Vs* anomaly that is observed. Simple calculations show that H_2_O bubbles with diameters of a few mm ascend through partially molten andesite at speeds of 1–3 meters per day, which is enough to move gas through the reservoir in a few years.

Changes of pressure in a volatile-saturated magma reservoir are set by the input/output budget^24^,^25^ (Supplementary material and Extended Data Figure S11). Positive contributions include magma replenishment from a deeper source and fractional crystallization, which acts to increase the volatile content of the residual melt and gas mixture. Negative contributions are due to the outflow of either magma or gas. Observations at NRV indicate a gradual increase of reservoir pressure (as indicated by deformation in and around the crater) accompanied by strong degassing until 2015. The 2015 magmatic eruption probably led to a decrease of reservoir pressure, which in turn promoted replenishment from the source. There is indeed evidence for a deeper reservoir beneath the NRV which seems to be connected to the shallow one^23^. The high *Vp/Vs* ratio that appears in the lower part of the reservoir in 2015–2016 is therefore likely due to a new batch of volatile-rich magma.

We suggest that this process may repeat itself periodically, with alternating phases of gas accumulation and release. Phases with the largest amounts of gas, which are associated with the strongest *Vp/Vs* anomalies, are likely to lead to magmatic eruptions.

## Additional Information

**How to cite this article**: Vargas, C. A. *et al*. Breathing of the Nevado del Ruiz volcano reservoir, Colombia, inferred from repeated seismic tomography. *Sci. Rep.* **7**, 46094; doi: 10.1038/srep46094 (2017).

**Publisher's note:** Springer Nature remains neutral with regard to jurisdictional claims in published maps and institutional affiliations.

## Supplementary Material

Supplementary Information
