# Supplementary material for: Breathing of the Nevado del Ruiz volcano reservoir, Colombia, inferred from repeated seismic tomography
Source: Sci Rep. 2017 Apr 10;7:46094. doi: 10.1038/srep46094 (PMC5385870; doi:10.1038/srep46094)
Supplement: Supplementary Information [file srep46094-s1.pdf]

Title: Breathing of the Nevado del Ruiz volcano reservoir, Colombia, inferred from repeated seismic tomography

Carlos. A. Vargas[1,*], Ivan Koulakov[2,3*], Claude Jaupart [4], Valery Gladkov[2,3], Eliana Gomez[1], Sami El Khrepy[5,6], and Nassir Al-Arifi[5]

Supplementary Information

This material includes more details on the activity of the NRV and additional explanation of the computation and testing of the tomography model.

The Recent activity of NRV

Since its November 1985 eruption, NRV has been the most dynamic of the active volcanoes in Colombia. Volcano-tectonic and long-period seismic events have followed a periodic pattern[6]. The current instrumental monitoring period, which began in 1985, has been characterized by recurring emissions of ash and volcanic gas. However, with the exception of two seismic crises that began in November 1995 and June 2002, episodes of high seismic activity were accompanied by magmatic eruptions that were observed by local inhabitants and that were detected by independent methods.

From May 2012 to the present, inclinometers near to main crater (Arenas) show deflation - inflation cycles superimposed on a continuous inflation trend in association with emissions of ash and SO_2 (Extended Data Figure S1). Significant tilt changes from -550 to 400 microradians record magma ascent and the subsequent emplacement of a dome in the eastern sector of the Arenas Crater[6]. This lava dome had superficial and thermal expression from September to November 2015. A mosaic of images of the COSMO-SkyMed radar from the Italian Space Agency illustrates the growth of the dome[6]. Flights over the crater confirmed the interaction of

dome material with the superficial structure. Drumbeat-type seismicity is associated with this phenomenon[7]. Among its most striking features is the similarity with low energy VT events, occurring with comparable waveforms and energy at relatively regular time intervals. Episodes of drumbeat seismicity of short duration are still detected today.

The observations are consistent with a simple model of sulfur degassing[23] based on (1) gas accumulation beneath an impermeable cap during times of repose, and (2) periodic gas release when the cap ruptures. The continued release of SO_2 after each eruption suggests that the cap is gradually released over several years.

In juvenile solid material emitted during the 1985 and 1989 eruptions of the NRV, Stix et al.[23] found evidence for pre-eruptive magma emplacement at shallow levels in (1) anhydrous mineral assemblages of plagioclase and pyroxene, (2) high silica contents of glasses, and (3) low water contents in melt inclusions (averaging between 1.6 and 3.3 wt.%). They proposed a multistage model of magma transport and degassing that involves alternating periods of magma ascent and magma ponding. Initially, volatile-bearing magma ascends from a deep reservoir located at depths of 9 – 15 km, driven by buoyancy. During decompression, the magma loses gas, particularly CO2 and sulfur. The magma eventually ponds at its neutral buoyancy level. Observations suggest a period of magma storage at shallow depths, where gas-saturated magma cools and crystallizes, thereby releasing gas. As a result, CO_2 is depleted from the residual melt whereas H_2O and SiO_2 are enriched due to fractional crystallization. H_2O enrichment is also due in part to increased solubility in the melt as CO_2 is degassed. Lastly, the density of the magma decreases as the level of dissolved H_2O increases, eventually causing the magma to become buoyant and to resume its ascent.

<u>Data and algorithms for repeated tomography</u>

In this study, we use the catalog of arrival times of P and S seismic waves from local events recorded by permanent seismic stations at Nevado del Ruiz Volcano (NRV). There are 57,646 events in the initial dataset, corresponding to 429,154 P-phases and 400,969 S-phases recorded by 124 seismic stations operated in the NRV area at different times. In this study, we used the data from 1998 onwards. Data from earlier times are available but were not used because they came from a relatively small number of stations.

The distributions of events for several time intervals are shown in Extended Data Figure 2. During the observation time, seismicity was largely concentrated beneath the summit area and started to migrate laterally towards the northwest and southwest in 2012.

We have selected data from four different time-intervals: 1998–2010, 2011–2012, 2013-2014, and 2015–2016, as indicated in Figure 1B by different colors. Here, we have considered the differences in the tomography results in periods 1, 3 and 4 with respect to results for the same period 2. This allowed us to avoid considerable differences in data distributions.

The data was selected to achieve maximum similarity of ray path distributions between the pairs of data subsets. The selection procedure was comprised of the following steps. (1) For the two-time episodes, we select one containing fewer data than another. (2) In this dataset, we range the events according to the number of picks per event from the maximum value, to the minimum one. (3) For the first event in the 1st dataset, we select all events in the 2nd dataset located at a distance less than a predefined value (0.5 km in our case). (4) For the selected events in the 2nd dataset, we count the number of common phases with the current event in the 1st subset (same stations and same types of wave, *P* or *S*). (5) We select one or several events having the maximum number of common phases. (6) In the case of several events with the same maximum number of common phases, we select one located at a minimum distance from the current event

in the 1st dataset. (7) The events are taken into consideration if the number of common phases is less than or equal to 8. The selected events are removed from both initial subsets. (8) The same procedure is repeated for the next event in the 1st subset. The numbers of the selected events and time pick in all cases are shown in Extended Data Table 1. In all cases, the total number of involved stations was 19.

The inversion was performed using a modified version of the LOTOS code[26]. The procedure starts with preliminary source locations using a 1D reference model and the grid search method. To speed up the preliminary location, we used straight ray paths to calculate travel times. The 1D reference model was the same as in the case of locating the entire dataset prior to data selection.

The iterative inversion procedure contains the recurrent steps of source locations in the 3D velocity model, matrix calculation, and the inversion. The source location procedure, in this case, uses the 3D bending algorithm for ray tracing[27].

The 3D distributions of the *P*- and *S*-wave velocities are parametrized by a set of nodes distributed in the study area according to the density of rays. Between the nodes, the velocity is approximated continuously using the tri-linear interpolation. The grids are constructed in the first iteration; then the velocity anomalies are updated for the same nodes. To reduce the effect of the grid geometry on the result, we performed the inversions for several grids with different basic orientations (0°, 22°, 45°, and 66° in our case), then computed the final model as the average of the resulting distributions. It is important that the grids be created for the first data subset; for the second subset, they are just copied from the first case.

The inversion was performed using the LSQR method[28,29]. We performed simultaneous inversions for the P- and S-velocity anomalies and source corrections (coordinates and origin

times). To stabilize the solution for the velocity distributions, we added two regularization matrices to the main matrix. One of them is diagonal, and it controls the amplitudes of the solutions in each parameterization node. Each row of the second matrix contains only two nonzero elements with the same values and opposite signs corresponding to all pairs of neighboring nodes in the grid. Changing the weight of this matrix leads to stronger or weaker flattening of the model.

We fixed the total number of iterations at three and further controlled the quality of the inversions by tuning the weights of the regularization matrices and source terms. The optimal values of the inversion parameters were determined according to the results of synthetic modeling, which are described below.

Synthetic modeling

An important part of any tomography study is synthetic modeling, which allows an assessment of the spatial resolution of the results and the determination of optimal values for the inversion parameters. In this study, synthetic modeling is necessary to distinguish between the impact of different ray path configurations and real velocity variations at depth.

The synthetic data were computed by three-dimensional ray tracing through a predefined synthetic model. In the LOTOS code, there are several possibilities for defining synthetic anomalies, both in map view and in vertical profiles. After calculation of synthetic travel times between the actual distribution of sources and receivers, the data were perturbed with random noise (0.02 and 0.05 s in our case) that provides the same variance reduction as in the case of the experimental data analysis. Any information about the source coordinates and origin times was "forgotten". The recovery procedure began with locating the events in the initial 1D model that

usually bias the sources significantly. The iterative inversion procedure and the inversion parameters used for recovering the synthetic model were identical to those for the experimental data.

To save space, we present the results of synthetic modeling for Series 1 only. For the other series, results appear to be similar and even better, because of a larger amount of data.

Extended Data Figure S3 shows the results of the checkerboard test, in which the starting model is composed of periodic anomalies with a size of 2.5 km and magnitude of ±8%. Signs for the P and S anomalies are opposite in order to generate large variations of the *Vp/Vs* ratio. With depth, the synthetic model remains unchanged. We performed an independent recovery of this model using the data subsets corresponding to time intervals 1998–2010 and 2011–2012. Results for both cases show that the main pattern is recovered (Extended Data Figure S3). In the bottom row, we present the difference between the recovered models. Deviations in the central part of the study area are less than 2% (note that the color scale interval for the difference is half that for the recovered models). A local instability in the S-velocity model is observed in the southwestern edge with a magnitude of ~4%, which we attribute to a few event mislocations and differences in the ray configurations in this part of the study area. Nevertheless, the changes in the model are significantly smaller than the amplitude of the main anomalies.

Another series of tests is presented in Extended Data Figure S4. In this case, we created synthetic models with realistic anomaly distributions in a vertical section, i.e. which are similar to those obtained using the real data. The anomalies were defined as polygons with unchanged shapes in the direction across the section. We defined the amplitudes of the P and S anomalies (pairs of numbers in each pattern) to ensure that the values of the *Vp/Vs* ratios are similar to those in the main experimental model. In the first case (middle column), we reconstructed identical

synthetic models (MODEL 1) using two data subsets corresponding to the time intervals of 1998–2010 and 2011–2012. We can see that the differences in the recovered models of *Vp/Vs* ratios are very small and do not exceed 0.03.

For the second case (right column in Extended Data Figure S4), we performed reconstructions of two considerably different models, 2 and 3. In particular, in Model 3, the value of the Vp/Vs deviation in the large anomaly at 2–3 km asl is significantly smaller than that in Model 2. The recovered results reveal robust changes between the models. This test is an important argument that this tomography scheme, which uses similar data configurations, does allow the retrieval of actual variations in the velocity structures. The calculated actual variations appear to be much more important than artifacts associated with noise and changes in ray distributions.

Inversion of experimental data

Based on the experimental data, we computed a total of six models, two for each series. In all cases, we performed three iterations and used the same inversion parameters. The numerical characteristics of the inversions are presented in Extended Data Table 1. It can be seen that the values of variance reduction computed in the L1 norm are relatively high compared to many tomography studies of similar scale in other volcanoes. For example, in the Klyuchevskoy volcano group[14], the variance reduction for S-data was not greater than 25%, whereas here it reaches 53%. This may be due to the high quality of the data in this study and clear strongly complex geological structures, which are well recovered by the tomography inversion. It can also be seen that the norm of the residuals gradually decreases with time. For example, the final average deviation of the S-residuals in 1998–2010 is 0.0678 s, while in 2015–2016 it is 0.0473 s.

The corresponding variance reductions are 43.05% and 53.74%, respectively. This may indicate the increase of data quality in more recent data compared to older data. The lower variance reduction for the first time interval may also be caused by changes in the velocity structure during this extended period.

The resulting P and S velocity anomalies and *Vp/Vs* ratios for three series are shown in horizontal and vertical sections in Extended Data Figures S5 to S10, in addition to the main result of the paper shown in Figure 3. For the first series of the time intervals of 1998–2010 and 2011–2012 (Extended Data Figure S5 and S6), we can see that the general shape of the anomalies remains consistent, especially for the *P* model. However, for the *S* model, we observe a considerable increase in the velocity. Such behavior of the *P* and *S* anomalies might indicate the migration of fluids, which does not affect the composition (*P* velocity), but strongly changes the shear modulus (*S* velocity).

In the vertical section, we see that beneath the volcano summit, the higher P velocity coexists with a strong negative S anomaly that results in a very high *Vp/Vs* ratio. In the first time interval, it exceeds 2.2, and in the second interval, it reaches 2.0. It should be noted that this anomaly of high *Vp/Vs* ratio matches a narrow, nearly vertical zone of seismicity just beneath the summit. The difference between the models in the first series shows that the structure is mostly changed by an increase of the S velocity to more than 10% beneath the summit at 2–3 km asl. This causes the corresponding decrease in the *Vp/Vs* ratio of more than 0.3.

It is important to compare the inversion results for the same interval of 2011–2012 derived for the three series. We see some differences that are especially prominent for the S-velocity distribution. These differences are merely due to changes in the data configurations. This indicates that comparing results without constructing identical datasets might be risky.

Interestingly, despite considerable differences in the distributions of the P and S anomalies, the models of *Vp/Vs* ratios look more similar in this case.

For the second series (Extended Data Figures S7 and S8), we observe a further decrease in the *Vp/Vs* ratio beneath the volcano. In the period 2013–2014, at 2–3 km asl, where we expect the magma reservoir to be located, the *Vp/Vs* ratio appears to be close to the average value in the model. Some anomalies of higher *Vp/Vs* ratios are observed in the deeper part of the model, which probably indicates the location of the conduit bringing fluids from deeper sources.

In the vertical sections corresponding to all time intervals, we observe a strong shallow anomaly of high *Vp/Vs* ratio in the summit area. This can be interpreted as strongly fractured, highly saturated rocks, which remain almost unchanged during the entire observation period.

In the case of both series, we observe considerable changes in the *P* and *S* anomalies and *Vp/Vs* ratios exceeding 10% in some places. These values are much stronger than the variations obtained in synthetic tests while recovering identical models. This observation implies that the changes in ray configurations, in this case, does not strongly affect the results, and the derived changes in both series represent actual changes in the Earth. These changes provide valuable information about the processes in the plumbing system beneath the Nevado del Ruiz volcano, as discussed in the main paper.

All the results presented in the paper can be reproduced using the data files and the program codes available at http://www.ivan-art.com/science/LOTOS/repeatomo.zip. This compressed file includes the Read_Me.pdf file with detailed guidelines on how to perform the calculations.

Numerical estimates for the magma sources

NRV is known for its very large venting of SO_2 gas and provides an excellent example of the general problem of the origin of volcanic gasses. In many volcanoes, it has proven to be difficult to reconcile the sulfur and magmatic budgets, such that the amount of sulfur that is lost is larger than what can be accounted for by magma[20]. Magma sulfur concentrations are determined from melt inclusion and glass data but, by definition, only provide values for magma that has already undergone crystallization. Also, it is known that sulfur solubility is low compared to that of H_2O, such that it may form gas at large pressures[22]. This has led to the conclusion that many magmas carry exsolved gas as they rise towards shallow storage zones[21]. Magma storage prior to the eruption, therefore, allows gas to escape and to get vented at the surface or through a near-surface hydrothermal system.

At NRV, melt inclusions and glass have highly variable silica, sulfur and water contents[23], indicating that volatile-saturated melt underwent fractional crystallization at different depths. The water content of volatile-saturated melt is dictated by the solubility law and varies as a function of pressure and CO_2 content. The lowest water content of 1.6 wt% provides an estimate of the shallowest depth of storage[23]. Depending on the CO_2 concentration, this depth is constrained to be at least 0.8 km (corresponding to zero CO_2 concentration). Similarly, the largest water content of 3.3 wt% indicates a minimum storage depth of 3.1 km[23]. Allowing for crystallization in a reservoir that is not a thin sill-like body, these two different estimates may be interpreted as indicative of the thickness of the shallow reservoir of Nevado del Ruiz. We note that these depths are consistent with our tomographic results which delineate a magma zone extending from about 2 to 4 km depth beneath the volcano summit.

We can also estimate the minimum magma volume using the amount of sulfur or water and assuming that it got degassed passively before the 2015 dome-building eruption. The total

mass of SO_2 gas erupted in the 2012-2015 period, over four years, is $7x10^6$ tons. The total amount of sulfur in subduction zone magmas has been reconstructed using phase equilibria relationships and data on both gas and melt[20], and is between 0.05 and 0.5 wt% with very rare exceptions. Using an average value of 0.1 wt%, we can estimate the amount of melt that is required to supply the mass of SO_2 gas that got vented. The amount of sulfur is $3.5x10^6$ tons, corresponding to a mass of melt of about $4x10^{12}$ kg. For an average density of andesite magma of 2600 kgm^{-3}, this corresponds to a volume of $1.4x10^9$ m^3 and an average diameter of about 1.4 km. An alternative calculation can be made using SO_2/H_2O ratios, which are typically between 2% and 5% by weight[20]. At the neighboring Galeras volcano, this ratio is 3%[20]. We can, therefore, convert the mass of SO_2 vented into one of $2.3x10^{11}$ kg for H_20. Assuming that magma entered the shallow reservoir with 3.3 wt% H_20 and that it progressively degassed to a concentration of 1.6 wt%[23], we obtain a magma volume of $5x10^9$ m^3 and a reservoir diameter of about 2 km. These estimates depend on various ratios that are not known precisely and are only accurate to within a factor of about 2. The orders of magnitude, however, are well constrained and lead to reservoir volumes that are consistent with our independent seismological estimate.

We can relate these observations to the melt budget of the reservoir. The total volume of the reservoir may change as a function of the reservoir average pressure P_R through deformation of the wall rocks. Changes of pressure induce changes of dissolved volatile content and density, such that one can define an effective compressibility β, which is much larger than that of the wall rocks[25]. The pressure change can be calculated as follows[24,25]:

$$V \beta \; dP_R/dt = Q_{in}/\rho - Q_{out}/\rho_g + Q_{cryst}$$

where V is the reservoir volume, P_R pressure, Q_{in} the mass flux of magma into the reservoir at density ρ, Q_{out} the mass flux of gas leaving the reservoir at density ρ_g. The last term on the right-hand side is:

$$Q_{cryst} = - V/\rho \, \partial\rho/\partial t \; dT/dt$$

where T is temperature, which is positive and represents a contribution due to crystallization, which acts to increase the volatile content of the residual melt and gas mixture. Assuming that there is no recharge (Q_{in}=0), for example, this equation has a steady-state solution with no change of pressure such that the amount of volatile that gets exsolved due to crystallization balances the amount of gas that gets vented out of the reservoir. A solution to this equation requires two closure relationships specifying how the inputs and outputs (Q_{in} and Q_{out}) vary as a function of reservoir pressure.

In an open conduit system connecting the shallow reservoir to a deeper magma source located at vertical distance h beneath the shallow reservoir (Extended Data Figure S11), the pressure difference driving the flow of magma into the reservoir can be written as:

$$\Delta P = P_S - P_R - \rho \, g \, h$$

where P_S is the source pressure. This shows that magma replenishment is enhanced by decreasing pressure in the shallow reservoir. It also shows that replenishment ceases when pressure in the shallow reservoir reaches a value equal to ($P_S - \rho \, g \, h$).

The driving pressure for gas venting, assuming permeable roof rocks, will always be positive and large owing to the small density of gas. Thus, the control on the rate of degassing is mostly due to the permeability, which is unknown, and to the availability of gas in the reservoir.

These simple considerations allow the following model for the 1998-2015 behavior of NRV. A large volatile-saturated magma batch emplaced in the shallow reservoir is degassing and

crystallizing. Gas bubbles rise through magma due to buoyancy, leading to a gas-depleted region that grows at the base of the reservoir. Simultaneously, crystallization leads to an increasing crystal content in the lower part of the reservoir. During that time, it is hard to predict changes of the reservoir pressure, which could be positive or negative depending on the respective magnitudes of the various terms in the pressure equation above. One important fact is that pressure changes are likely to be small due to the contribution of crystallization (i.e. Q_{cryst}) and to the large value of compressibility in a gas-rich reservoir. The 2015 eruption implied the rapid loss of a significant mass and volume from the reservoir, which led to decrease of the reservoir pressure P_R. One of the consequences, as shown by the driving pressure equation, is replenishment of the reservoir by magma from the deeper source. This is consistent with a change of *Vp/Vs* in the deeper part of the storage zone, which can be interpreted as due to a batch of new magma.

Degassing of the reservoir is fed by gas bubbles rising through the reservoir. With time, as stated above, one expects that a degassed region grows at the base of the reservoir. If one can track the rise of the boundary between the gas-depleted lower region and the gas-rich upper region, one can estimate an average ascent rate for gas bubbles. According to our tomographic images, this rate is about 1 km per 4 years or about 10^{-5} ms^{-1}. Using the well-known formula for the velocity of gas bubbles through melt:

$$V = 1/3\ (a^2\ \rho\ g)/\ \mu$$

where a is the bubble radius, ρ the melt density, μ the melt viscosity and where we have assumed that the density and viscosity of gas are very much smaller than those of melt. For volatile-saturated andesitic melt at the base of the reservoir with 3.3 wt% dissolved H_2O, the viscosity is about 10^4 Pas[23]. For the velocity estimate of 10^{-5} ms^{-1}, bubbles with 3 mm radii are required.

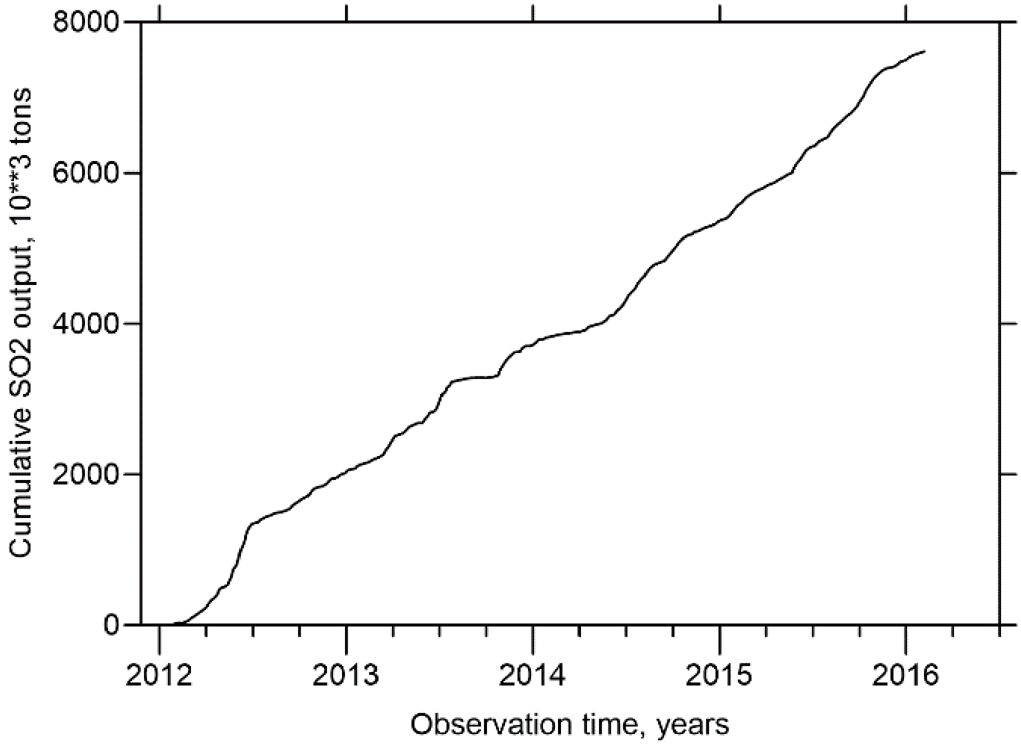

Extended Data Figure S1. Graph of the cumulative output of SO_2 from NRV measured by the Manizalez Volcanological Observatory[6].

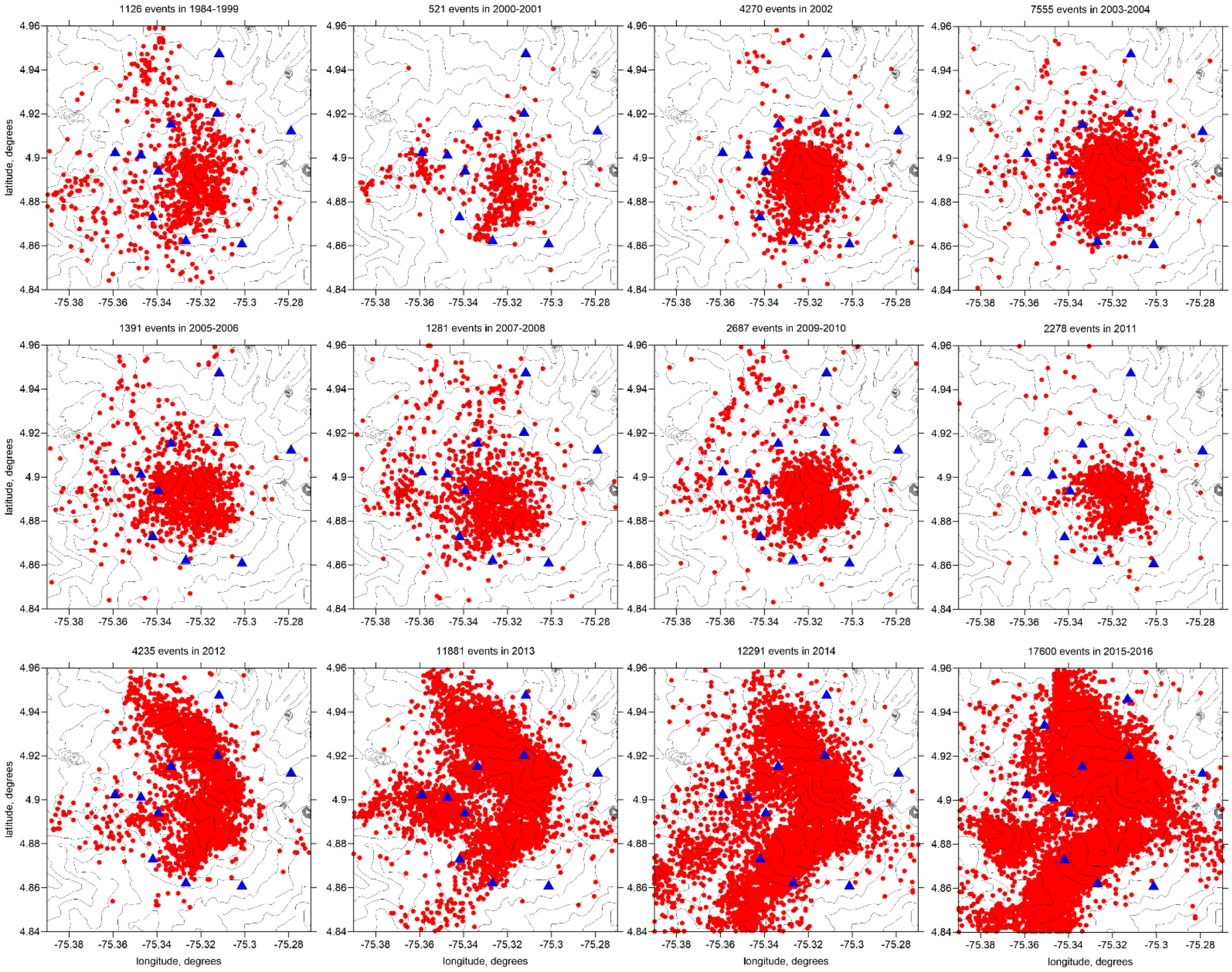

Extended Data Figure S2. The distributions of events in different times of observations. The time of the interval and the number of events are indicated above each plot. The red dots are the earthquakes, and the blue triangles are the seismic stations. Contour lines depict the relief[30]. This picture is produced using Surfer 12, Golden Software[31].

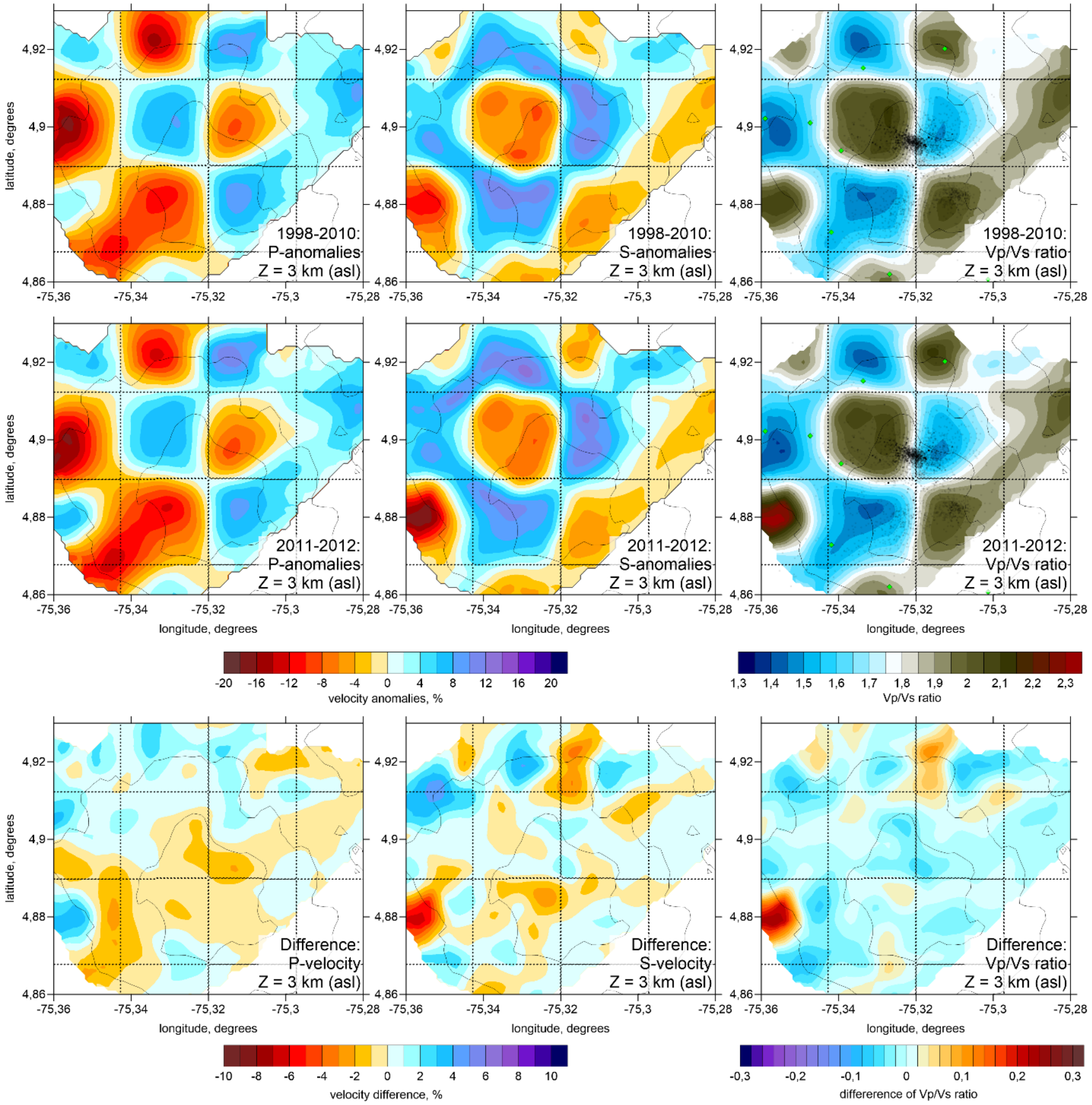

Extended Data Figure S3. An example of the checkerboard test for the Series 1. Upper two rows show the inversion results at the altitude of 3 km for the P- and S anomalies and Vp/Vs ratio. The lower row is the difference between these models. The shapes of the initial synthetic anomalies are depicted with dotted lines. The contour lines depict the relief[30]. Note that the color scale intervals for the anomalies and differences are different. This picture is produced using Surfer 12, Golden Software[31].

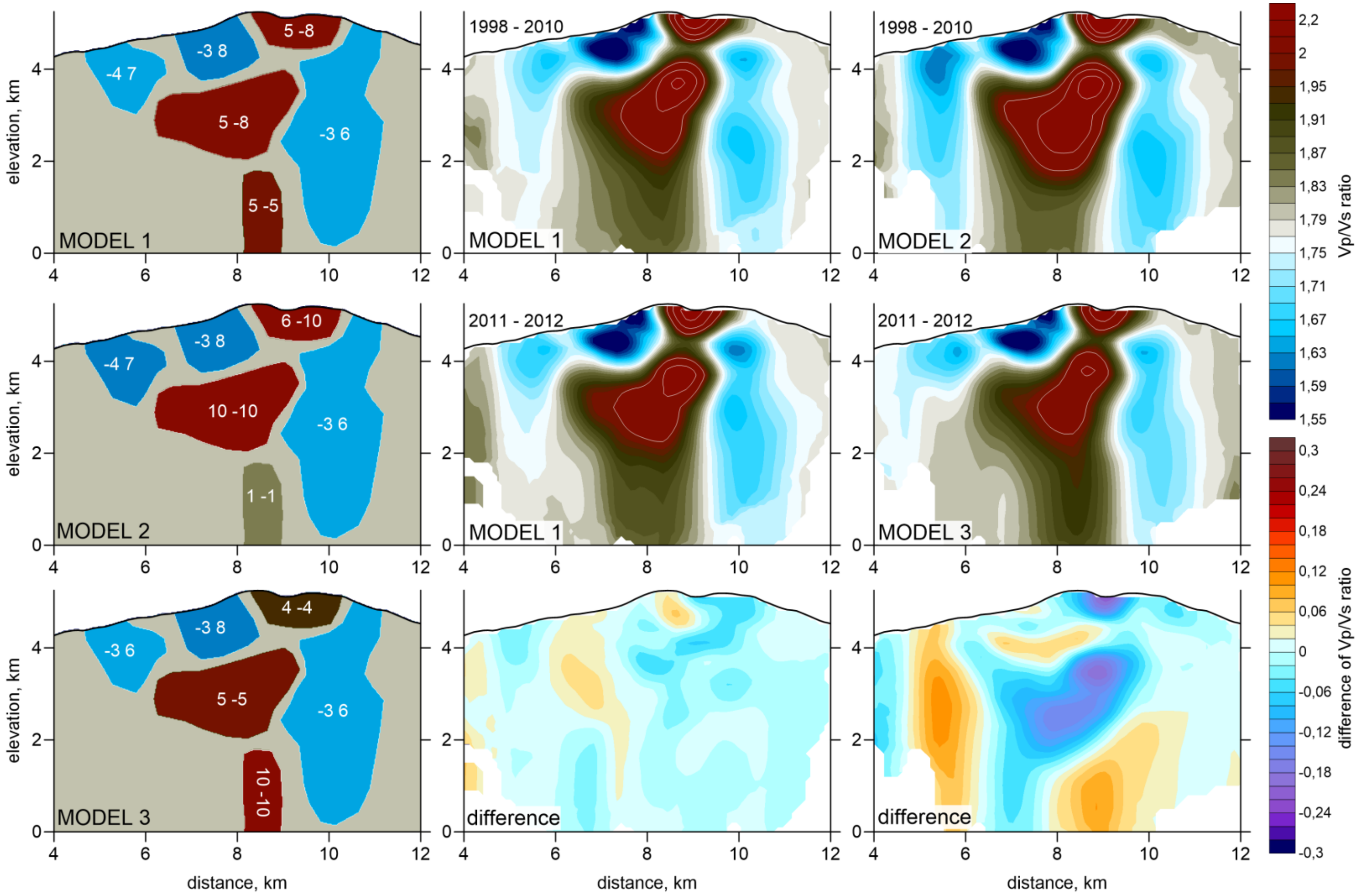

Extended Data Figure S4. Results of modeling the repeated tomography for the Series 1 with synthetic anomalies of realistic shape. Left column presents the shapes of the initial anomalies of Vp/Vs ratio for three different models. Number pairs indicate the values of P and S anomalies in percent. Middle row is the recovering results in the case of the same synthetic Model 1 and difference (lower plot). Right column presents the reconstruction results and the difference between two different models 2 and 3. This picture is produced using Surfer 12, Golden Software[31].

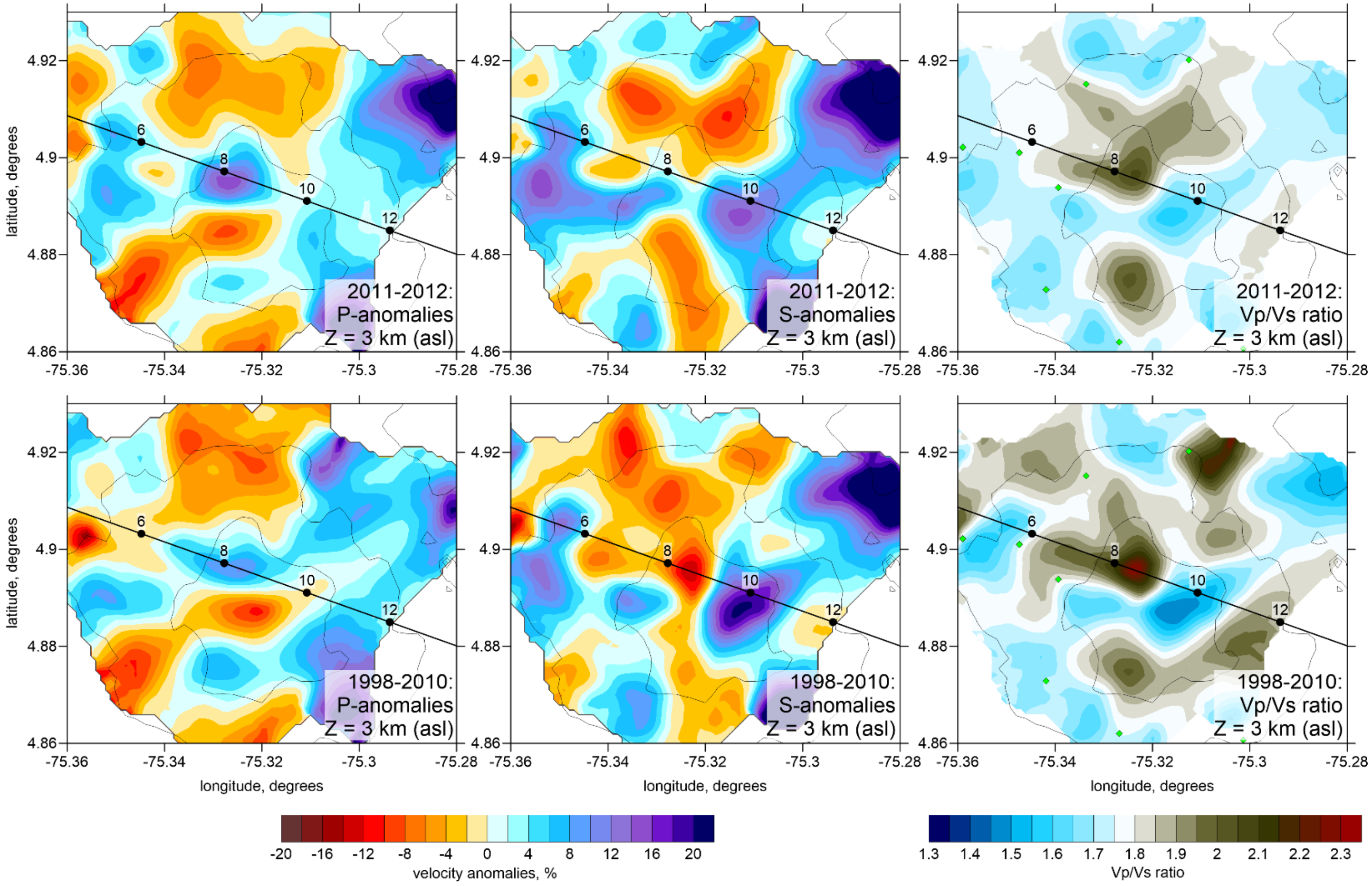

Extended Data Figure S5. Results of experimental data inversions for two-time intervals corresponding to the series 1 at the altitude of 3 km asl. The distributions of the P and S anomalies and Vp/Vs ratios are presented. The line indicates the location of the vertical section used for presenting the main results. The contour lines depict the relief[30]. Note that the color scale intervals for the anomalies and differences are different. This picture is produced using Surfer 12, Golden Software[31].

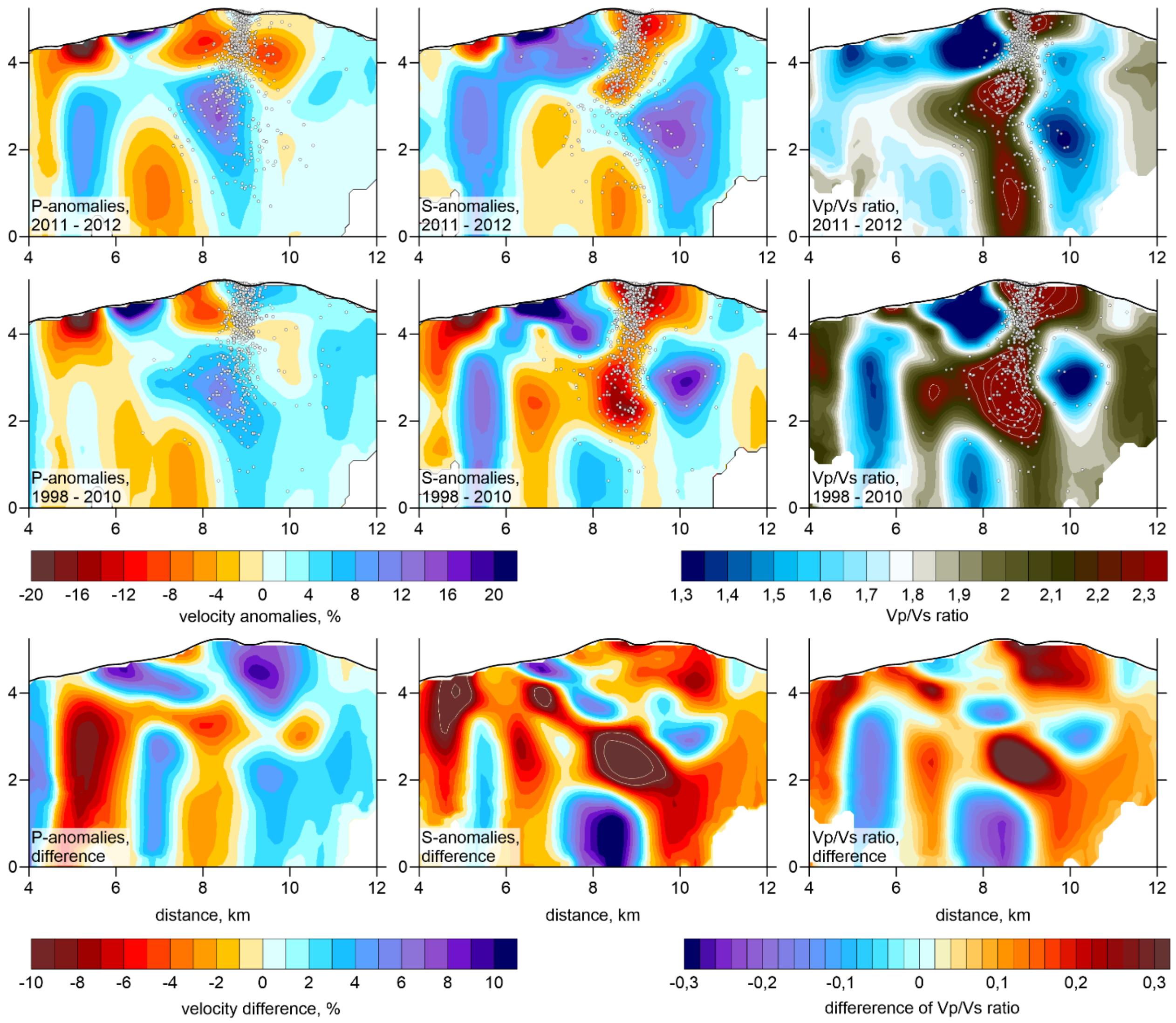

Extended Data Figure S6. Results of experimental data inversions for two-time intervals corresponding to the series 1 in the vertical section. The distributions of the P and S anomalies and Vp/Vs ratios and their differences are presented. The dots depict the earthquakes located at distances of less than 0.4 km from the profile. This picture is produced using Surfer 12, Golden Software[31].

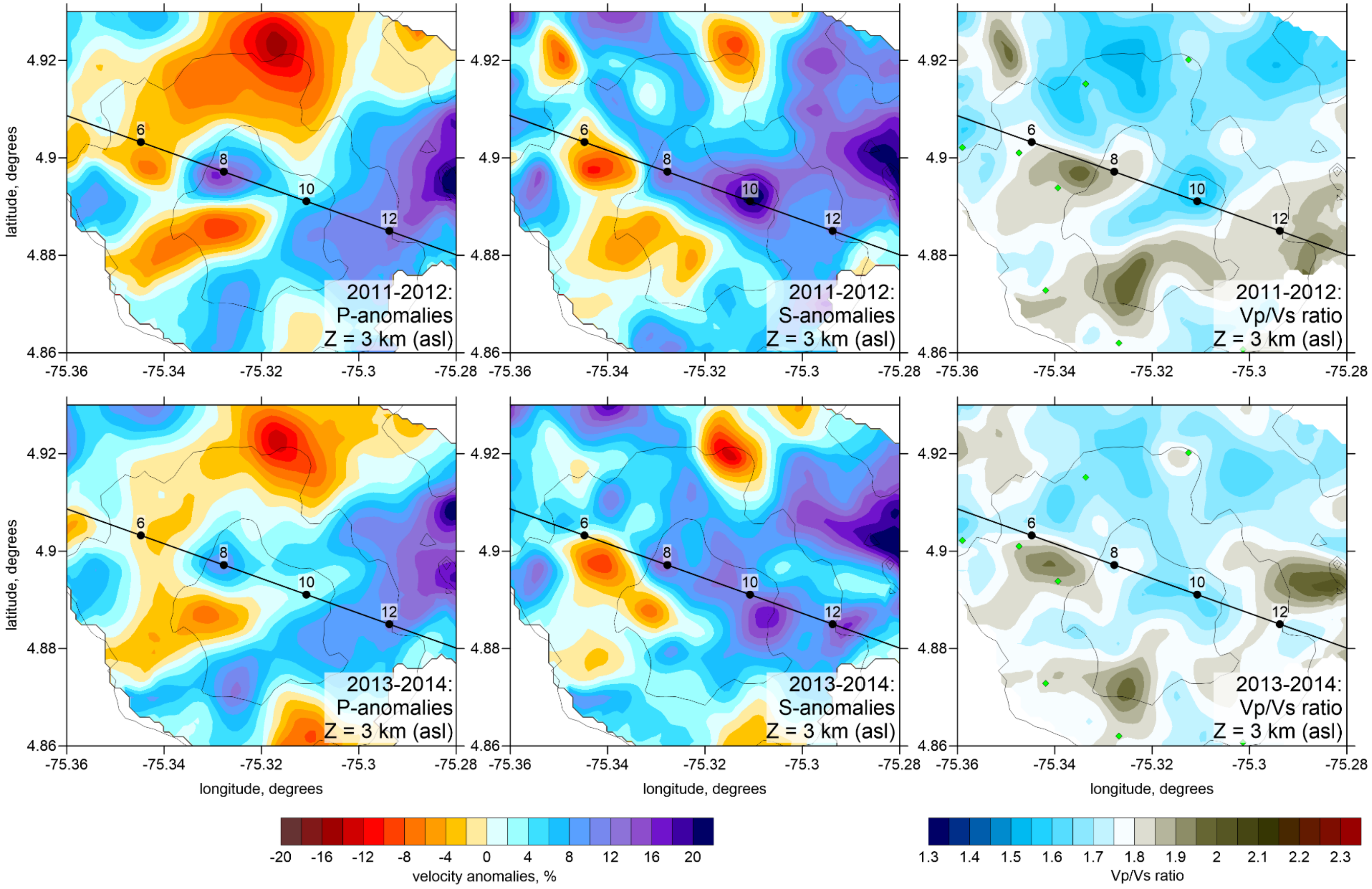

Extended Data Figure S7. Same as Extended Data Figure S6, but for the Series 2.

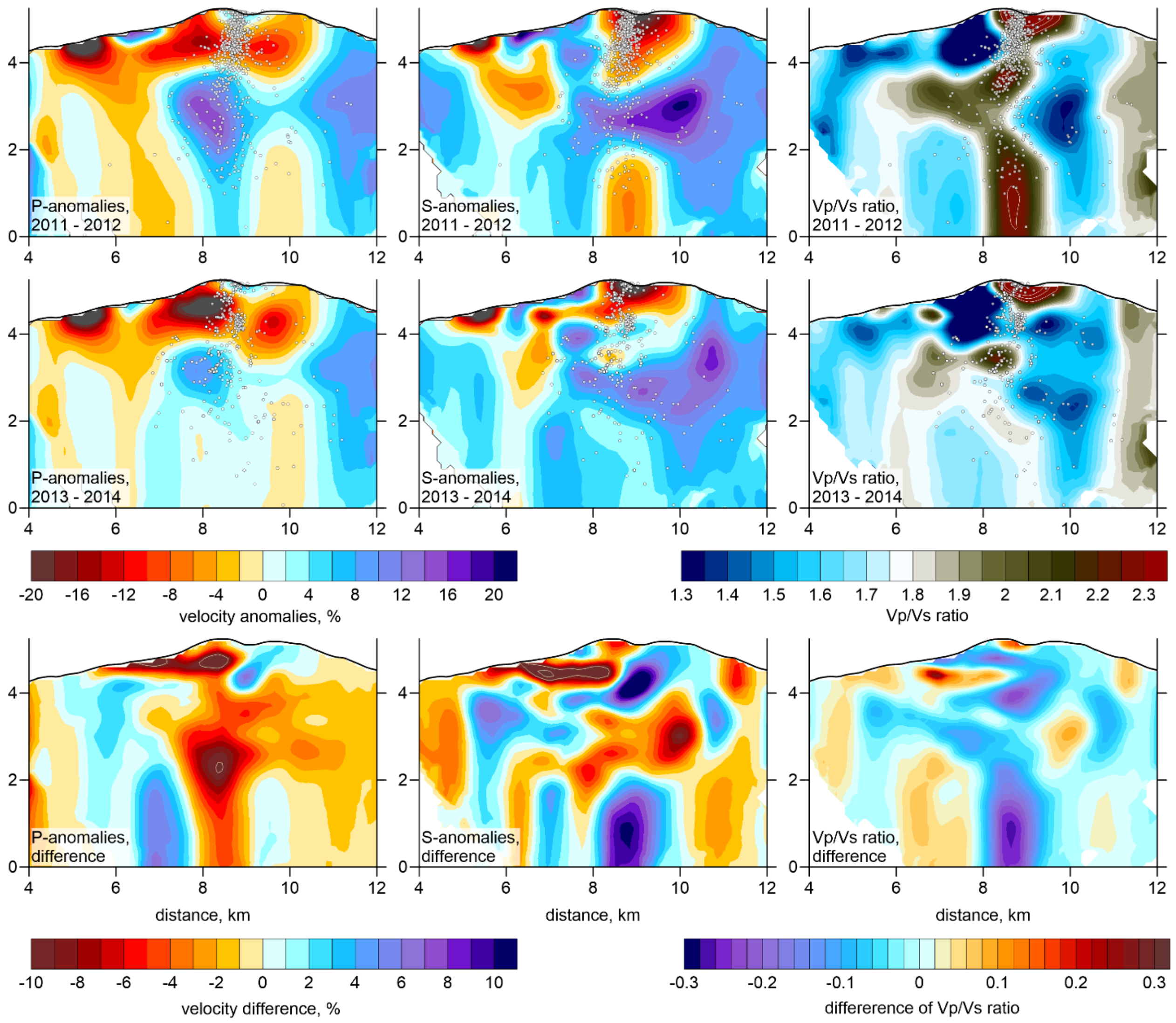

Extended Data Figure S8. Same as Extended Data Figure S7, but for the Series 2.

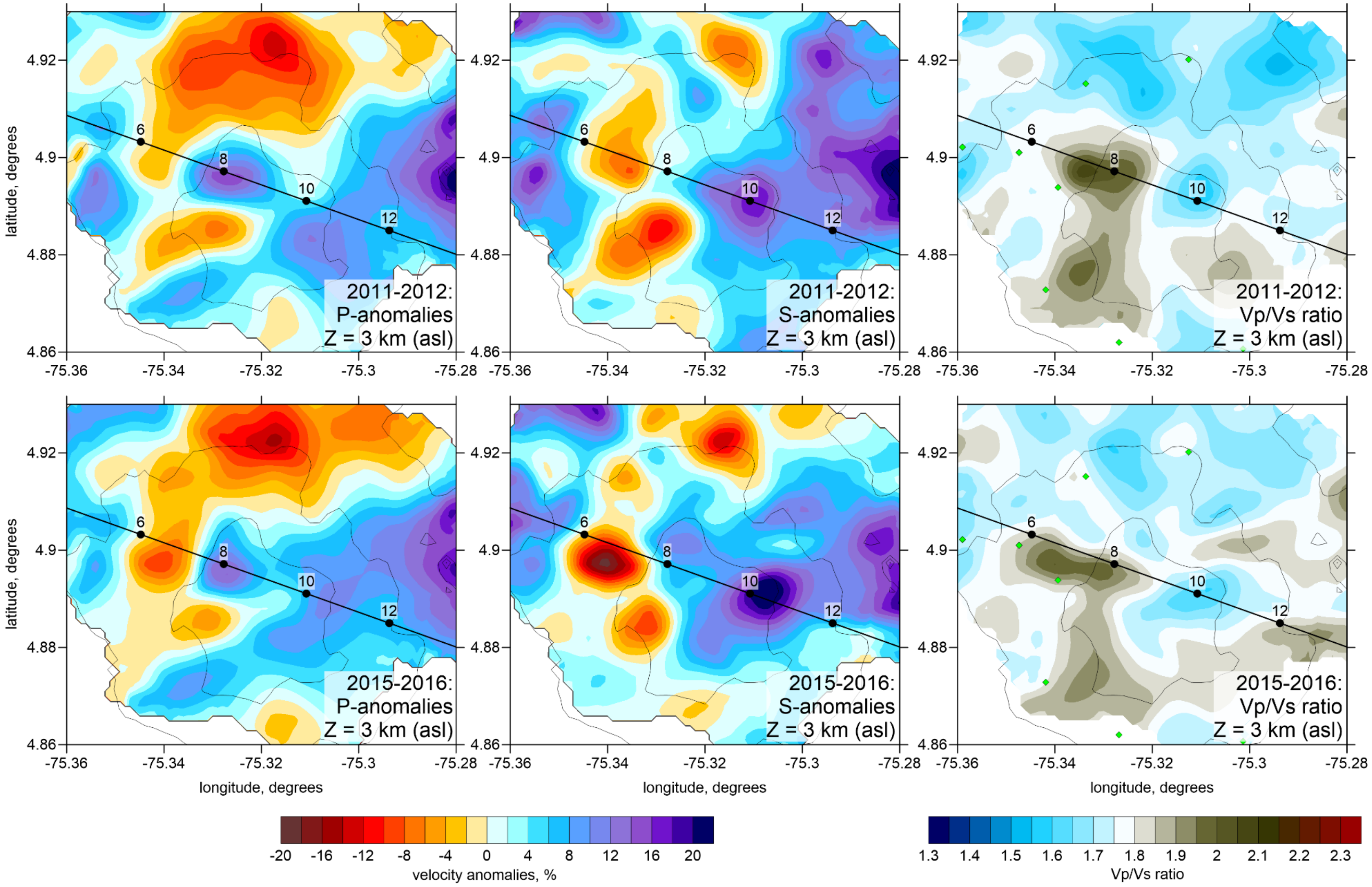

Extended Data Figure S9. Same as Extended Data Figure S6, but for the Series 3.

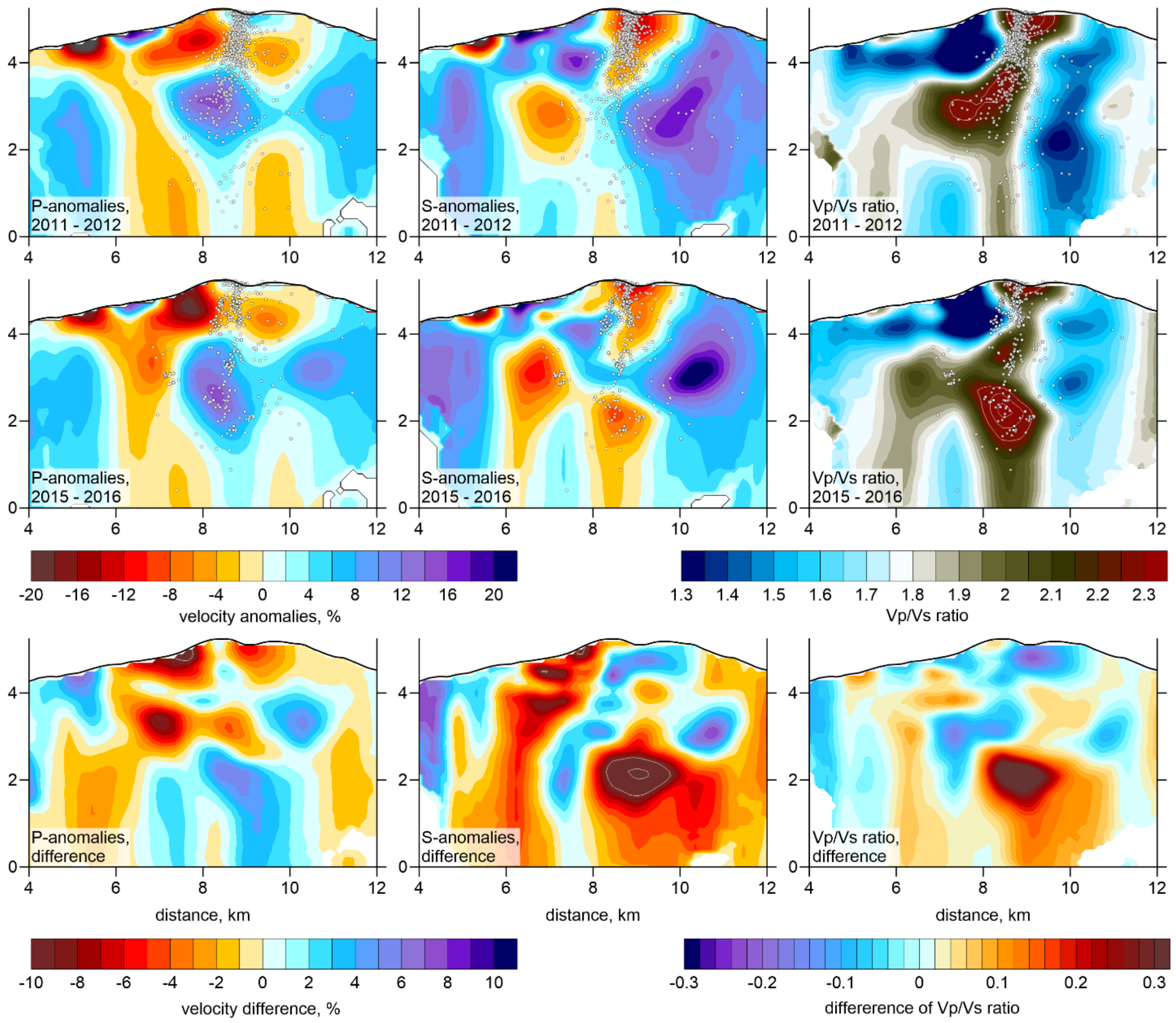

Extended Data Figure S10. Same as Extended Data Figure S7, but for the Series 3.

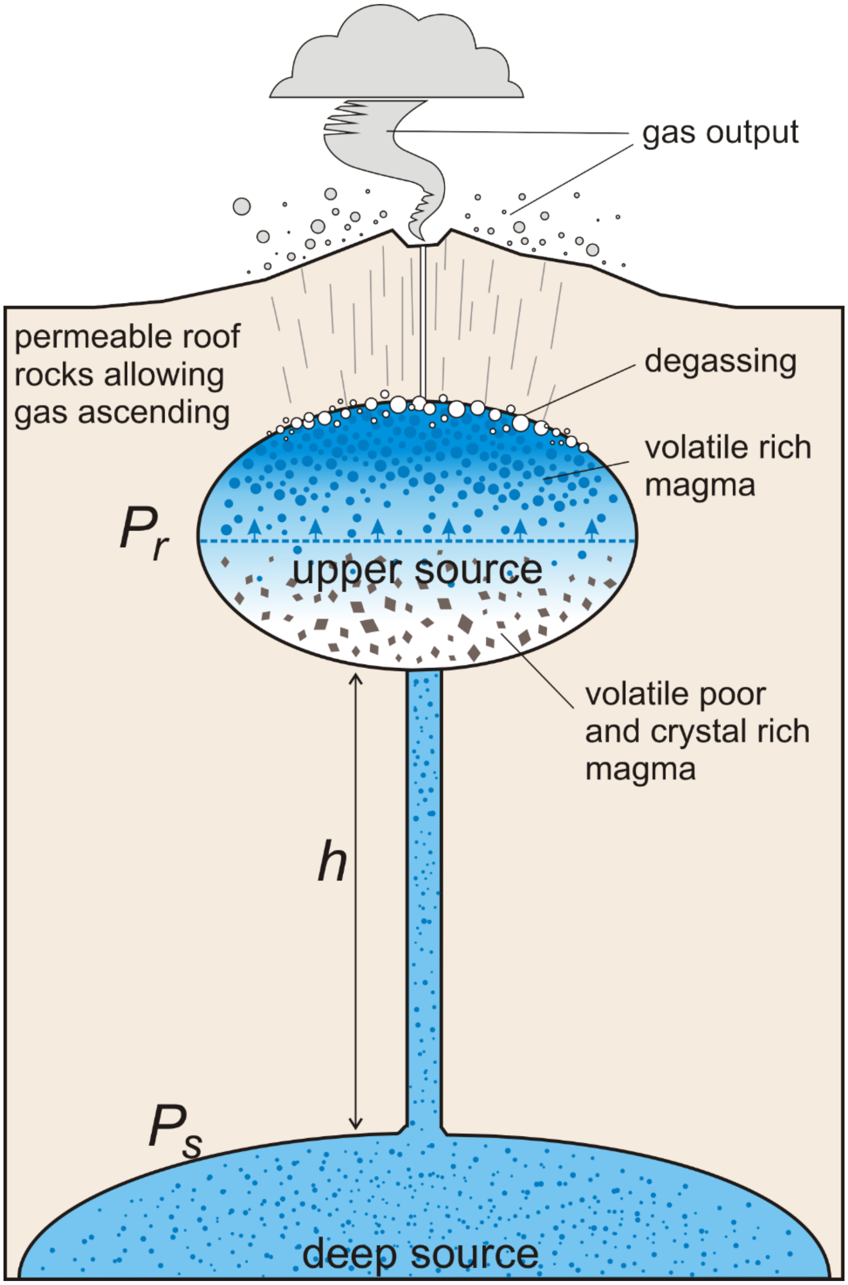

Extended Data Figure S11. Simplified scheme of magma sources used for numerical estimates in the text.

Extended Data Table 1. Information about numbers of data and values of residuals for two series of repeated tomography inversions

Series	Time intervals	Number of events	Data type:	Number of rays	Starting residual, s	Final residual, s	Residual reduction, %
Series 1	2011-2012	4487	P-data:	28725	0.0722	0.0512	33.64
			S-data	26747	0.1127	0.0653	41.99
	1998-2010	4487	P-data:	28725	0.0789	0.0522	33.86
			S-data	26747	0.1192	0.0678	43.05
Series 2	2011-2012	6036	P-data:	44051	0.0830	0.0556	39.38
			S-data	41712	0.1323	0.0749	49.76
	2013-2014	6036	P-data:	44051	0.0717	0.0396	44.69
			S-data	41712	0.1195	0.0555	53.51
Series 3	2011-2012	5913	P-data:	38862	0.0801	0.0498	37.78
			S-data	36865	0.1233	0.0639	48.12
	2015-2016	5913	P-data:	38862	0.0625	0.0352	43.65
			S-data	36865	0.1024	0.0473	53.79
